# Assessment of Adherence to Oral Iron Supplementation Guidelines in Patients With Iron Deficiency Anemia: A Cross-Sectional Study

**DOI:** 10.7759/cureus.87681

**Published:** 2025-07-10

**Authors:** Ambreen Ambreen, Aleeza Habib, Ali Hassan Sajjad, Ali Jawad Malik, Qasim Javed

**Affiliations:** 1 Internal Medicine, Shifa International Hospital Faisalabad, Faisalabad, PAK; 2 Internal Medicine, Gujranwala Medical College, Gujranwala, PAK; 3 Internal Medicine, Shaikh Khalifa Bin Zayed Al-Nahyan Medical and Dental College, Lahore, PAK; 4 Internal Medicine, Central Park Medical College, Lahore, PAK; 5 Nephrology, Shifa International Hospital Faisalabad, Faisalabad, PAK

**Keywords:** adherence, clinical audit, iron deficiency anemia, oral iron therapy, patient compliance, prescribing practices

## Abstract

Background and objective

The most common kind of anemia in the world is iron deficiency anemia (IDA), and following recommendations for oral iron supplements is still crucial to the effectiveness of therapy. The main objective of this study is to assess the level of adherence to oral iron supplementation guidelines among patients with IDA, focusing on prescribing practices, patient compliance, and follow-up monitoring in a clinical setting.

Methodology

A two-phase cross-sectional clinical audit was conducted in the Outpatient Medical Departments of Gujranwala Medical College and Allied Teaching Hospitals, Gujranwala, Pakistan. Convenience sampling was used to recruit 278 adult individuals with an IDA diagnosis who were provided oral iron treatment. From January to June 2023, Phase 1 (n = 139) was carried out. This was followed by an intervention phase that included patient information leaflets and physician education. The second phase (n = 139) took place between October 2023 and March 2024. A 10-point audit proforma was used to evaluate adherence, with scores classified as excellent (≥8), moderate (5-7), or poor (≤4). IBM SPSS Statistics for Windows, Version 25 (released 2017; IBM Corp., Armonk, NY, USA) was used to analyze the data, and a paired-sample t-test was used to assess statistical significance.

Results

Good adherence was shown by 41 out of 139 patients (29.50%) in Phase 1 and 79 out of 139 patients (56.83%) in Phase 2. The number of patients with poor adherence dropped from 34 (24.46%) to 16 (11.51%). There was a substantial improvement in the mean adherence score, from 5.43 ± 1.78 to 7.26 ± 1.45 (p < 0.001). In Phase 2, 131 patients (94.24%) received written teaching materials, but in Phase 1, none were provided. Follow-up monitoring, including hemoglobin/ferritin reassessment, rose from 63 patients (45.32%) to 117 patients (84.17%).

Conclusions

Targeted educational interventions significantly improved adherence to oral iron supplementation guidelines among patients with IDA.

## Introduction

The most prevalent kind of anemia in the world, iron deficiency anemia (IDA), affects both industrialized and developing nations [1,2]. It is caused by a lack of iron available for the synthesis of hemoglobin, which is often brought on by a poor diet, prolonged blood loss, malabsorption, or an increase in physiological demand [3,4].

Clinical guidelines recommend specific doses, formulations, and durations of oral iron supplementation to optimize absorption and minimize side effects, making it the standard first-line therapy for IDA [5,6]. However, adherence to oral iron treatment remains a persistent challenge in clinical practice despite these standardized recommendations [7].

Nonadherence is influenced by a combination of systemic, provider, and patient-related factors. Common patient-related barriers include gastrointestinal side effects, misunderstanding of dosing instructions, and underestimation of the need to continue therapy after symptom improvement [8,9]. At the provider level, deviations from guidelines may stem from clinical judgment, time constraints for counseling, or lack of familiarity with updated recommendations [10]. System-level factors, such as limited follow-up and medication availability, further exacerbate adherence challenges, leading to poor outcomes like chronic anemia, increased healthcare utilization, and reduced quality of life [11].

Although adherence to oral iron therapy is crucial for the successful management of IDA, there is limited real-world evidence systematically evaluating compliance with guideline-directed therapy, particularly in outpatient settings, where long-term treatment is typically administered. Existing studies often focus narrowly on patient-reported compliance, overlooking provider prescribing patterns and follow-up monitoring. This lack of comprehensive audits in outpatient clinical practice represents a critical gap in the literature. Addressing this gap can help identify actionable areas for improvement and inform targeted interventions.

Therefore, this study aimed to conduct a real-world clinical audit to evaluate adherence to oral iron supplementation guidelines among patients with IDA in an outpatient setting, focusing on prescribing practices, patient compliance, and follow-up monitoring, and to assess the impact of targeted educational interventions on improving these outcomes.

## Materials and methods

Study design and setting

This two-phase cross-sectional clinical audit was conducted in the Outpatient Medical Departments of Gujranwala Medical College and Allied Teaching Hospitals, Gujranwala, Pakistan. The audit included 278 adult patients diagnosed with IDA and prescribed oral iron supplements. Convenience sampling was used to recruit patients presenting during the audit periods, which limits generalizability and is acknowledged as a study limitation.

Inclusion and exclusion criteria

Patients ≥18 years of age who were prescribed oral iron therapy after diagnosis of IDA based on clinical and laboratory findings were included. Patients were excluded if they were pregnant (due to different supplementation guidelines), receiving intravenous iron or blood transfusions, had anemia of other causes (e.g., chronic disease or hemolysis), or were unable to provide informed consent.

Sampling and sample size

The sample size was determined by feasibility over the two predefined six-month audit cycles, while adhering to accepted audit standards recommending at least 20-50 cases per cycle for outpatient quality improvement initiatives [12]. A larger sample of 139 patients per phase was chosen to enhance benchmarking reliability and detect meaningful practice changes. Given the descriptive, observational nature of the study and the lack of hypothesis testing, no formal power calculation was performed [13]. The chosen sample size was considered sufficient to identify clinically relevant trends in adherence.

Audit tool and data collection

The audit proforma was developed collaboratively with clinical experts to assess adherence across three domains: follow-up monitoring (including hemoglobin/ferritin reassessment and reinforcement of recommendations), patient compliance (timing with meals, consistency, and side effect management), and prescribing practices (appropriate formulation, dose, and frequency). The proforma was pilot-tested on 10 patients for feasibility and reviewed by three independent clinicians for face and content validity. Each question was scored as “Yes” (1) or “No” (0), with overall scores categorized as high (≥8), moderate (5-7), or poor (≤4) adherence. The treating physician, anonymized to the auditor, completed the proforma at follow-up visits, while patient compliance was confirmed verbally. Proformas were collected biweekly from a secure collection point. The full audit proforma is provided in Appendix A.

Audit phases

Two distinct phases were conducted: Phase 1 (Initial Audit, January-June 2023) collected baseline data from 139 patients. This was followed by a three-month intervention period (July-September 2023), after which Phase 2 (Re-audit, October 2023-March 2024) assessed a new cohort of 139 patients using the same methodology. The same group of physicians participated in both phases to minimize provider-related confounding, while different patient cohorts reduced carryover effects.

Educational intervention

The remedial intervention implemented after Phase 1 consisted of two interactive educational sessions delivered in person by consultant hematologists and senior pharmacists. The sessions covered updated IDA management guidelines, strategies for patient counseling, and recommended follow-up monitoring practices. Printed pamphlets summarizing these points were also distributed to physicians (Appendix B). Concurrently, patients were given information leaflets emphasizing adherence, side-effect management, and the importance of follow-up (Appendix C).

Statistical analysis

Data were analyzed using IBM SPSS Statistics for Windows, Version 25 (released 2017; IBM Corp., Armonk, NY, USA). Descriptive statistics summarized patient characteristics and adherence scores. Comparisons between Phase 1 and Phase 2 adherence scores were made using paired-sample t-tests, with p-values < 0.05 considered statistically significant.

Ethical considerations

Ethical approval was obtained from the Institutional Review Board (IRB) of Gujranwala Medical College and Allied Teaching Hospitals (approval no. ME.IRB.416/GMC). Verbal informed consent was obtained from all participants, consistent with local IRB guidance for minimal-risk, observational quality improvement activities. Patient identity and physician performance data remained confidential.

## Results

The baseline characteristics of the 278 patients who were part of the study are shown in Table 1. Of these, 86 patients (30.94%) were between the ages of 18 and 30, 121 patients (43.53%) were between the ages of 31 and 50, and 71 patients (25.54%) were older than 50. There were 176 females (63.31%) and 102 males (36.69%) in the sample. In terms of symptom duration, 72 patients (25.90%) reported symptoms lasting less than a month, 125 patients (44.96%) reported one to three months, and 81 patients (29.14%) reported more than three months. 

**Table 1:** Baseline Characteristics of Patients (n = 278)

Variable Category	Variable	Number of Patients (n, %)
Age Group (Years)	18-30	86 (30.94)
	31-50	121 (43.53)
	>50	71 (25.54)
Gender	Male	102 (36.69)
	Female	176 (63.31)
Duration of Symptoms	<1 month	72 (25.90)
	1-3 months	125 (44.96)
	>3 months	81 (29.14)

**Figure 1:**
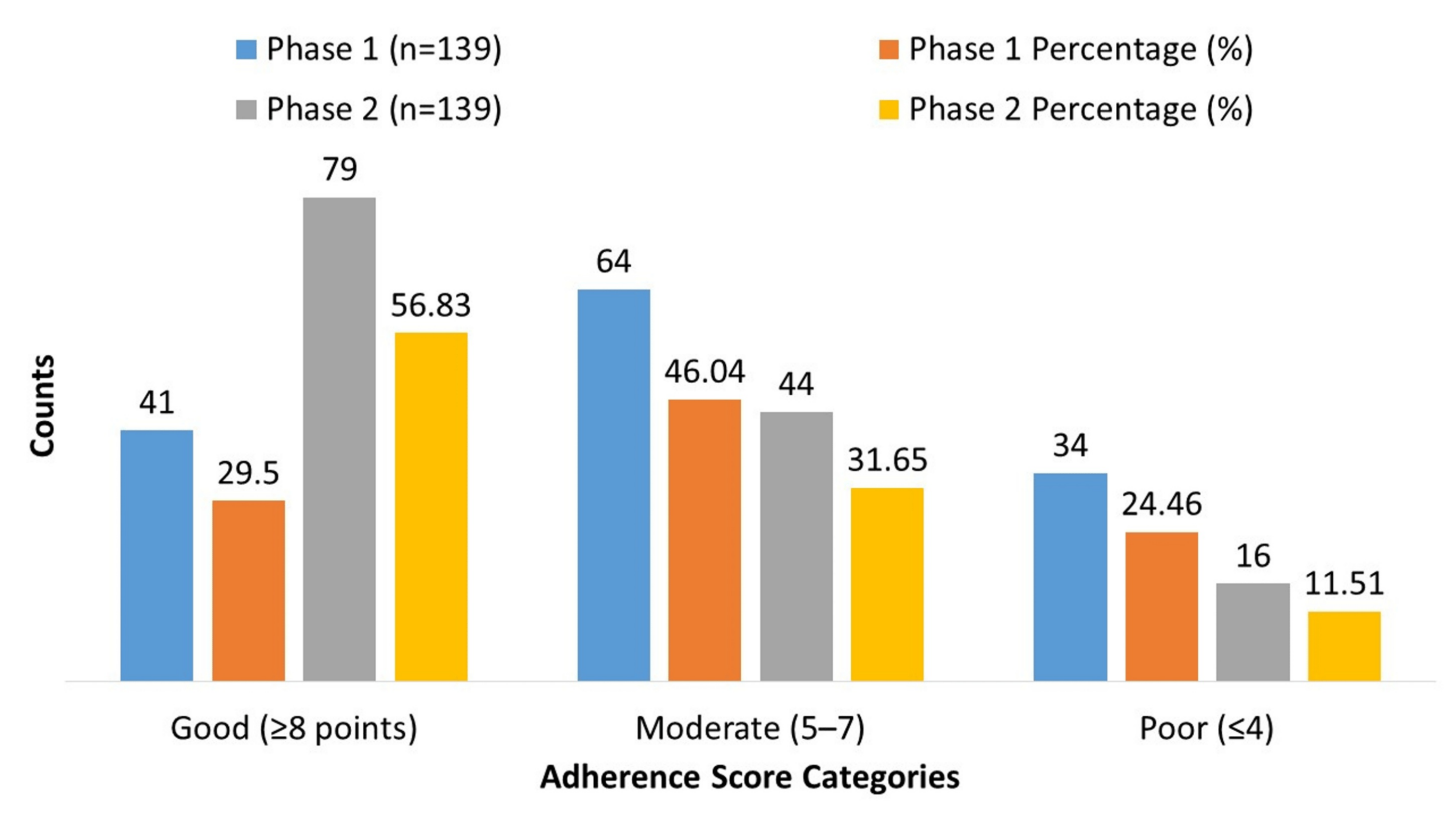
Adherence Score Categories - Phase 1 vs. Phase 2 A chi-square (χ²) test for trend showed a value of 28.4, with a p-value < 0.001, indicating a statistically significant improvement in adherence distribution.

Phase 1 and Phase 2 adherence score groups, each consisting of 139 patients, are contrasted in Figure 1. In Phase 1, 34 patients (24.46%) had poor adherence, 64 patients (46.04%) had moderate adherence, and 41 patients (29.50%) had strong adherence. Following the Phase 2 intervention, there was a significant improvement in adherence behavior, with excellent adherence rising to 79 patients (56.83%), moderate adherence falling to 44 patients (31.65%), and poor adherence decreasing to 16 patients (11.51%). A chi-square test showed a significant improvement in adherence category distribution between Phase 1 and Phase 2.

**Figure 2:**
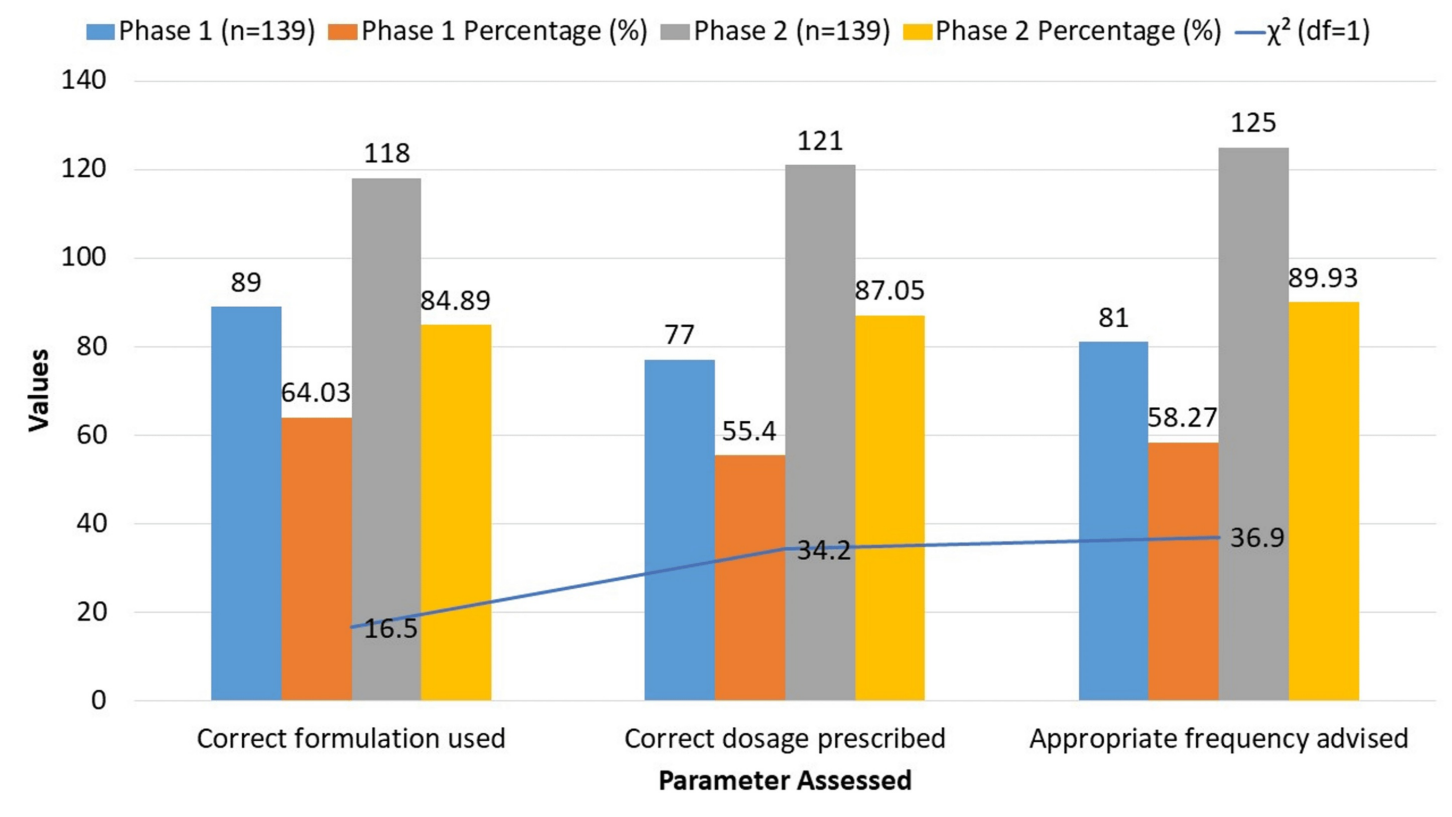
Prescribing Practices Assessment The p-value for each Prescribing Practices Assessment was < 0.001. A p-value < 0.05 was considered statistically significant. χ²: chi-square; df: degrees of freedom

Prescription procedures evaluated in both periods are shown in Figure 2. In Phase 1, 89 patients (64.03%) received the correct formulation, 77 patients (55.40%) received the correct dose, and 81 patients (57.27%) received the correct frequency instructions. In Phase 2, these metrics improved: 118 patients (84.89%) received the correct formulation, 121 patients (87.05%) received the correct dose, and 125 patients (89.93%) received the correct frequency. Chi-square tests demonstrated significant improvements in prescribing practices between phases.

**Figure 3:**
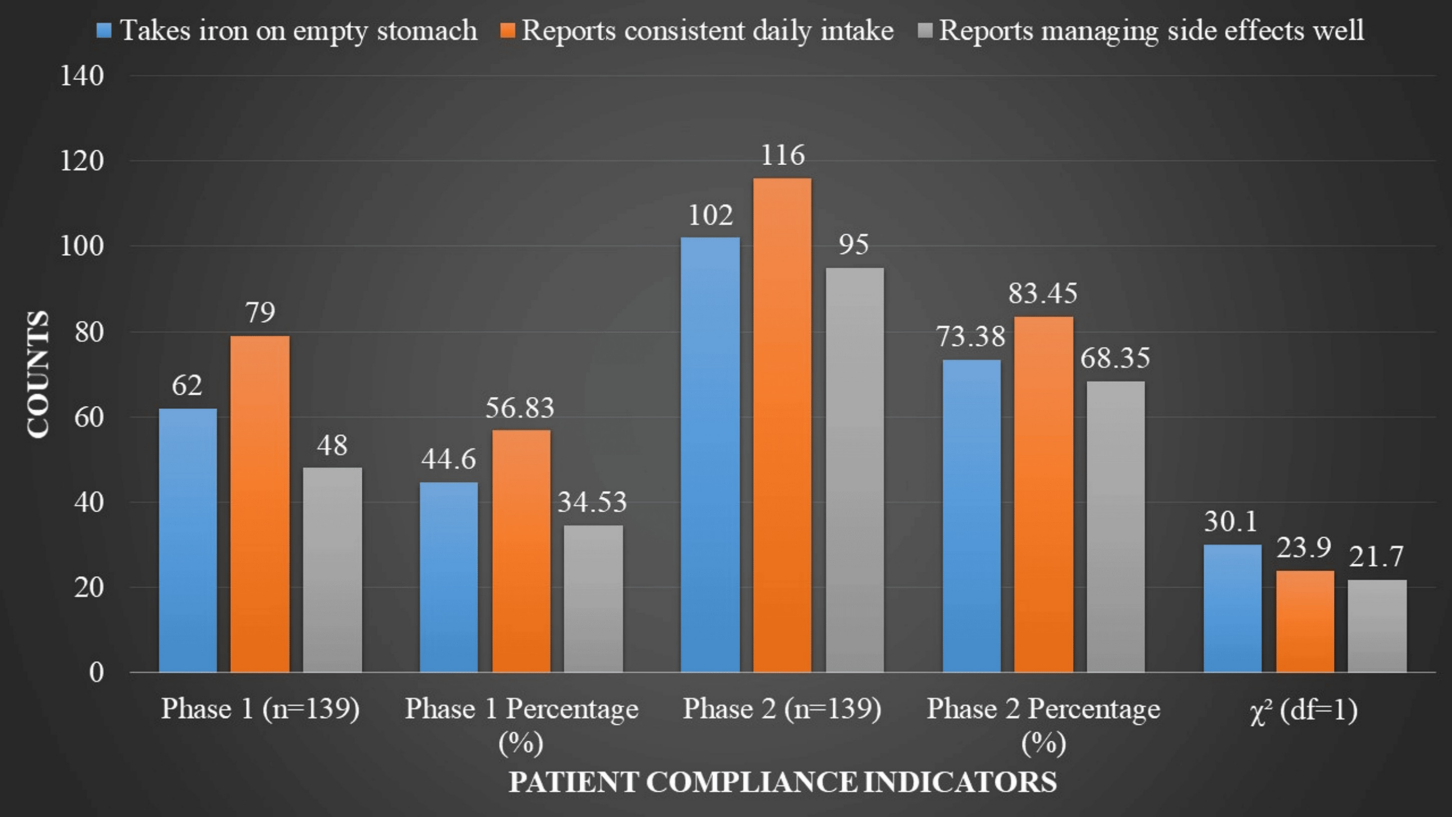
Patient Compliance Indicators The p-value for each Prescribing Practices Assessment was < 0.001. A p-value < 0.05 was considered statistically significant. χ²: chi-square; df: degrees of freedom

Indicators of patient compliance are highlighted in Figure 3. In Phase 1, 48 patients (34.53%) effectively controlled adverse effects, 79 patients (56.83%) reported consistent daily intake, and 62 patients (44.60%) reported taking iron on an empty stomach. Phase 2 showed a significant improvement in compliance, with 95 patients (68.35%) successfully managing adverse effects, 116 patients (83.45%) adhering daily, and 102 patients (73.38%) taking iron as prescribed. Chi-square tests revealed significant gains in compliance indicators between phases - specifically, managing side effects, daily adherence, and taking iron on an empty stomach.

The educational interventions and follow-up monitoring are presented in Table 2. In Phase 1, 54 patients (38.85%) received counseling, 63 patients (45.32%) had their hemoglobin/ferritin levels evaluated, and none received written material. However, Phase 2 demonstrated significant progress, with 117 patients (84.17%) receiving a reassessment, 122 (87.77%) receiving counseling, and 131 (94.24%) receiving written teaching materials. Chi-square tests confirmed significant improvements in follow-up and education.

**Table 2:** Follow-Up Monitoring and Education (Phase 1 vs. Phase 2) *p < 0.05 considered statistically significant. χ²: chi-square; df: degrees of freedom

Parameter Assessed	Phase 1, n (%)	Phase 2, n (%)	χ² (df = 1)	p-value*
Reassessment of hemoglobin/ferritin	63 (45.3)	117 (84.2)	43.3	<0.001
Counseling on iron therapy	54 (38.9)	122 (87.8)	66.1	<0.001
Written information leaflets	0 (0.0)	131 (94.2)	192.0	<0.001

A paired-sample t-test was used in Table 3 to compare mean adherence scores. Among 139 patients in each phase, the mean adherence score in Phase 1 was 5.43 (SD ± 1.78), whereas in Phase 2, it increased dramatically to 7.26 (SD ± 1.45). After the intervention, there was a statistically significant improvement in adherence. The paired-sample t-test indicated a significant increase in mean adherence score from Phase 1 to Phase 2 (t(138) = 11.5, p < 0.001, 95% CI: 1.46-2.19).

**Table 3:** Comparison of Mean Adherence Scores Before and After Intervention (Paired-Sample t-test) *p < 0.05 considered statistically significant.

Phase; N	Mean Adherence Score	Standard Deviation (SD)	Mean Difference (95% CI)	t-value	p-value
Phase 1 (Pre-intervention); 139	5.43	1.78	1.83 (1.46-2.19)	11.2	<0.001*
Phase 2 (Post-intervention); 139	7.26	1.45			

## Discussion

This audit demonstrated that targeted educational interventions significantly improved adherence to oral iron supplementation guidelines, encompassing physician prescribing practices, patient compliance, and follow-up monitoring. The proportion of patients with high adherence nearly doubled after the intervention, while poor adherence declined by more than half. These findings underscore the value of structured education programs in addressing the multifaceted barriers to effective anemia management, consistent with prior evidence showing that provider- and patient-focused initiatives enhance adherence to iron therapy [14]. Importantly, these improvements were not uniform across all components, suggesting that some aspects of adherence may be more amenable to change than others.

Frequency adherence and provision of written information leaflets showed particularly marked gains post-intervention, likely because these aspects are easier to standardize and reinforce through visual aids and clear protocols. In contrast, while managing adverse effects improved, it remained lower than other domains, reflecting the persistent challenge of tolerability issues associated with oral iron therapy. Similar obstacles were highlighted in prior studies, where gastrointestinal side effects accounted for over 40% of patient discontinuations [15]. These findings highlight the importance of sustained patient counseling, proactive management of side effects, and consideration of alternative formulations when intolerance persists.

The substantial improvements in physician prescribing practices align with reports from similar audits that have identified significant gaps between clinical guidelines and real-world practice [16,17]. Educational reinforcement appears effective in reducing such variability by updating clinicians on best practices and clarifying the rationale behind guideline recommendations. In our study, correct prescribing of formulation, dose, and frequency improved significantly following the intervention, highlighting how targeted clinician education can translate into better patient care.

Improvements in patient education and follow-up monitoring were particularly noteworthy. Nearly all Phase 2 patients received written information leaflets, and rates of counseling and laboratory reassessment more than doubled compared to baseline. These enhancements are in line with accepted clinical best practices, which emphasize proactive patient education and regular monitoring to ensure accurate diagnosis, sustained treatment compliance, and optimal management of IDA [18]. The use of written materials may have facilitated better recall and self-management, providing a low-cost, scalable strategy to improve care quality and outcomes.

The overall success of the multimodal interventions was reflected in the significant rise in mean adherence scores, from 5.43 ± 1.78 at baseline to 7.26 ± 1.45 after intervention (p < 0.001). This supports findings from earlier systematic reviews, which reported that patient-centered approaches - particularly those incorporating patient-reported outcomes and education - enhance therapeutic engagement and adherence in the management of anemia [19]. These findings have broader implications: structured, multimodal education strategies - combining clinician training, patient materials, and regular monitoring - can feasibly improve adherence in outpatient settings and may be adapted to other chronic conditions requiring long-term therapy. Future research should explore the durability of these improvements over longer periods and assess their applicability in diverse healthcare contexts, including primary care and rural settings.

Strengths and limitations

This study has several notable strengths. The two-phase audit design enabled a robust assessment of both baseline adherence to oral iron supplementation guidelines and the impact of targeted educational interventions over time, reflecting real-world clinical practice. The use of a structured, pilot-tested, and expert-validated audit proforma ensured reliable and consistent data collection across multiple domains, while the combined evaluation of physician prescribing practices, patient-reported compliance, and follow-up monitoring provided a comprehensive and nuanced understanding of adherence behaviors. Furthermore, the inclusion of educational materials for both physicians and patients enhances the transparency and replicability of our intervention strategy.

However, some limitations warrant consideration. The use of convenience sampling may restrict the generalizability of our findings to other settings with differing patient populations or healthcare resources. Nonetheless, this pragmatic sampling reflects typical outpatient clinic workflows and strengthens the study’s relevance to routine care. Patient-reported compliance data are inherently subject to recall and social desirability biases, although we mitigated this by having treating physicians verbally confirm key responses. We did not assess the underlying etiology of IDA, focusing instead on adherence behaviors common to standard oral iron therapy, regardless of cause. Finally, as a single-center audit, our findings may not fully represent practices in primary care or rural settings, and long-term adherence beyond the audit period was not assessed. These limitations highlight opportunities for future multi-center, longer-term studies to validate and extend our findings.

However, several limitations should be acknowledged. The use of convenience sampling may limit generalizability, particularly to settings with different patient demographics or healthcare resources. Patient compliance was assessed through self-reporting, which is subject to recall bias and social desirability effects. The single-center nature of the study may not fully reflect practices in primary care or rural settings. Finally, longer-term adherence beyond the audit period was not evaluated.

## Conclusions

People with IDA did not follow the oral iron supplement guidelines well at the start, but this improved significantly after they received specific education and monitoring. Prescription practices, patient compliance behaviors, and follow-up monitoring all showed marked improvements; the mean adherence score increased, and the proportion of patients with excellent adherence rose. These findings highlight the importance of structured patient counseling, physician education, and adherence to guideline-based care in optimizing the management of IDA.

Looking forward, these results also suggest that integrating structured, multimodal education strategies into routine clinical practice could inform the development of updated guidelines that emphasize patient-centered education, written materials, and regular monitoring as standard components of care. Future research should evaluate the long-term sustainability, cost-effectiveness, and scalability of such interventions in diverse settings, including primary care, rural healthcare, and low-resource environments. Such efforts could support broader implementation of evidence-based practices and contribute to reducing the global burden of IDA through improved adherence and outcomes.

## Appendices

Appendix A

Audit Proforma: Assessment of Adherence to Oral Iron Supplementation Guidelines in IDA Patients

Patient ID: ___________ Age: ___________ Gender: ☐ Male ☐ Female Date of Visit: ___________

Section A: Prescribing Practices (Max: 3 points)

Item

Yes (1)

No (0)

1. Correct formulation of iron prescribed (e.g., ferrous sulfate, ferrous fumarate)

☐

☐

2. Appropriate dosage according to body weight/standard adult dose

☐

☐

3. Appropriate frequency (e.g., once or twice daily as per guidelines)

☐

☐

Section B: Patient Compliance (Max: 3 points)

Item

Yes (1)

No (0)

4. Takes iron on an empty stomach or as advised

☐

☐

5. Reports consistent daily intake over the last week

☐

☐

6. Manages side effects (e.g., nausea, constipation) effectively

☐

☐

Section C: Follow-Up Monitoring and Education (Max: 4 points)

Item

Yes (1)

No (0)

7. Hemoglobin and/or ferritin reassessed after 4-6 weeks

☐

☐

8. Reinforcement of adherence advice provided verbally

☐

☐

9. Written educational material/leaflet given

☐

☐

10. Informed about duration and signs of treatment response

☐

☐

Total Score (out of 10): ___________

Adherence Category: ☐ Good (≥8 points) ☐ Moderate (5-7 points) ☐ Poor (≤4 points)

Completed By (initials only): _______ Date Submitted: ___________

Appendix B

**Table 4:** Physician Education Material Summary

Topic	Content
Objective	Reinforce guideline-recommended prescribing, monitoring, and counseling practices for oral iron therapy in IDA.
Key Topics Covered	Overview of IDA: causes, epidemiology, health impact
	Recommended formulations, doses, and frequencies
	Duration of therapy and when to switch to IV iron
	Addressing nonadherence: managing side effects
	Counseling techniques to improve adherence
	Monitoring: timing of follow-up labs
	Reinforcing adherence during each visit
Delivery Format	Two 1-hour interactive in-person sessions
	Case examples and Q&A
Facilitators	Consultant hematologist
	Senior pharmacist
Materials Provided	Slide presentation
	One-page quick-reference summary

Appendix C

**Table 5:** Patient Information Leaflet Content

Section	Content
How to Take Iron Medicine	Take on an empty stomach if tolerated
	If upset stomach, take with light food
	Avoid tea, coffee, milk, and high-fiber foods at the same time
	Take at the same time every day
	Do not stop when you feel better - complete the full course
Common Side Effects	Nausea
	Constipation
	Dark stools
	Mild stomach cramps
	Usually improve with time
	Contact doctor if severe
Follow-Up	Blood tests (hemoglobin and ferritin) in 4-6 weeks
	Attend all follow-up appointments
Why Adherence Matters	Missing doses delays recovery
	Stopping early may worsen anemia
	Always ask questions if unsure
